# Elucidating the surface geometric design of hydrophobic Australian *Eucalyptus* leaves: experimental and modeling studies

**DOI:** 10.1016/j.heliyon.2019.e01316

**Published:** 2019-03-18

**Authors:** Hua Guo, Zonghan Xie, Jeremy Shaw, Kingsley Dixon, Zhong-Tao Jiang, Chun-Yang Yin, Xuemei Liu

**Affiliations:** a School of Engineering, Edith Cowan University, Joondalup, WA 6027, Australia; b School of Mechanical Engineering, The University of Adelaide, Adelaide, SA 5005, Australia; c Centre for Microscopy, Characterization & Analysis, The University of Western Australia, Crawley, WA 6009, Australia; d School of Molecular and Life Sciences, Curtin University, Bentley, WA 6102, Australia; e School of Engineering & Information Technology, Murdoch University, Murdoch, WA 6150, Australia; f Newcastle University in Singapore, SIT Building@Ngee Ann Polytechnic, 537 Clementi Road #06-01, Clementi, 599493, Singapore; g Department of Infrastructure Engineering, The University of Melbourne, Parkville, VIC 3010, Australia

**Keywords:** Structural biology, Plant biology

## Abstract

Three Australian native *Eucalyptus* species, i.e., *Eucalyptus woodwardii*, *Eucalyptus pachyphylla* and *Eucalyptus dolorosa*, were investigated, for the first time, with respect to the hydrophobicity of their leaves. It is well established that these leaves exhibit exceptionally high water repellency, in addition to an extraordinary ability to retain water, albeit their specific wetting mechanisms are still poorly understood. To identify the critical factors underlying this phenomenon, the surface topography of these leaves was subjected to micro-examination (SEM). Micro- and nanometer scale surface roughness was revealed, resembling that of the quintessential “lotus effect”. Surface free energy analysis was performed on two models based on the surface topographies of the study *Eucalyptus* species and lotus, in order to study wetting transitions on these specific microscopic surface features. The influence of surface geometrical parameters, such as edge-to-edge distance, base radius and cylindrical height, on surface free energy with different liquid penetration depths was studied with these two models. Larger energy barriers and smaller liquid-solid contact areas were more influential in the calculations for the lotus than for *Eucalyptus*. The information obtained from these two models may be useful for guiding the design of novel artificial surfaces in the collection and transport of micro-volume liquids.

## 1 Introduction

Many biological surfaces, such as plant leaves, bird feathers and animal furs, exhibit strong water repellency in order to adapt to environmental conditions. A typical example is the adaxial surface of the lotus leaf *Nelumbo nucifera*, which remains completely dry while floating on water. The wetting characteristics of the lotus leaf include high contact angles and low contact angle hysteresis [1, 2, 3, 4], attributed largely to a hierarchical surface structure. These properties have formed the basis of many surface-critical applications; for example, self-cleaning [5, 6, 7], corrosion prevention [8], drag reduction [9] and fouling control [10]. Recently, high contact angle hysteresis was observed for certain rose petals exhibiting high contacts angles [11]. These petals contain micro-bumps with relatively wide spacing and small peak-to-base heights. Consequently, a composite wetting scenario prevails – that is, the Wenzel process [12] is dominant on the micro-structure level, allowing water to enter the asperities, therefore leading to a high contact area and high adhesion; and the Cassie mode [13], rules on the nano-structure level, maintaining high water contact angles. Such unique wetting behavior may lead to applications in mass and heat transport [14] or microfluidic devices [15].

The contact angle, commonly used to quantify the wettability of a solid surface, is a function of two independent variables: the surface energy of the solid controlled by its chemical composition and bonding states, and the surface roughness. Evidence has shown that the presence and three-dimensional (3D) geometry of surface patterns can markedly alter the wetting responses of a surface. For example, micro-structured features, in the form of pores and posts on a leaf surface, can increase water contact angles substantially. Recent studies revealed that nano-structured roughness can further enhance the water repellency and help maintain a robust composite wetting [11, 16, 17, 18, 19]. Therefore, understanding the relationships between surface geometric parameters and wetting properties may assist in the development of new types of surfaces with enhanced integrated characteristics.

*Eucalyptus*, a plant species found widespread across temperate, arid and tropical ecosystems in Australia, is well adapted for coping with environmental extremes. It is widely noticed that *Eucalyptus* leaves, especially those shiny juvenile leaves, present considerable water repellency with water forming spheres on the leaf surface and the droplets tend to adhere to the leaf surface even when the leaf is turned upside down. This is a sharp contrast compared to the lotus leaf, on which water forms perfect spheres that roll off readily even when the leaf is slightly tilted. The special wettability of *Eucalyptus* leaves may inspire and trigger relevant applications other than superhydrophobicity or self-cleaning. However, the wetting properties of *Eucalyptus* leaves have never been studied in detail, with only an investigation of surface leaf waxes undertaken in 1970 of 315 *Eucalyptus* species for taxonomic purposes [20]. Here, three Australian native *Eucalyptus* species with a prominent waxy-white leaf form were comprehensively studied with emphasis on elucidating the link between the surface micro-structures and leaf surface hydrophobicity. In view of this, physical models were proposed based on the dimensions of surface micro-structure features observed on their leaves. Surface energy analysis was carried out on two physical models to compare wetting scenario transition between the *Eucalyptus* species and the lotus *Nelumbo nucifera* leaf. Energy barrier and energy potential were quantitatively identified during the speculated wetting transition process.

## 2 Experimental

### 2.1 Sample preparation

Fresh leaves of three *Eucalyptus* species, i.e., *E. woodwardii*, *E. pachyphylla*, and *E. dolorosa* were collected from specimens growing in the nursery at the Western Australian Botanic garden in Kings Park. Squares of 100 mm^2^ were carefully excised from the abaxial side of these leaves for optical observation and contact angle measurements. Smaller squares of 4 mm^2^ cut from these leaves were freeze-dried prior to SEM examination using an Emitech K775X Turbo Freeze Dryer (Quorum Technologies Ltd, Kent, UK). The following time-temperature path was adopted: holding at −120 °C for first 5 min, then gradually rising to −65 °C in a period of 14 hours slowly increasing to room temperature over 10 hours.

To study the influence of nano-structural wax on the wettability of leaf surfaces, leaf samples without wax were also prepared. Square specimens of 100 mm^2^ were firstly cut from fresh *Eucalyptus* leaves, followed by immersing in 200 mL of chloroform for 15 min to dissolve the wax layer from the specimen surface. The specimens were then washed three times with 5 mL of chloroform and dried in a fume hood for 2 hours to evaporate the solvent prior to wettability measurements.

### 2.2 Instrumentation

The contact angle measurements were performed at ambient temperatures using an FTA 1000 Drop Shape Analysis system (First Ten Angstroms Inc., Virginia, USA) equipped with an automated dispensing syringe and a computer-controlled tilt stage. To ensure an even surface for water dispersal, leaf samples were fixed on the stage by double-sided adhesive tape before the application of 5 *μ*L water droplets. The average water contact angles (*n* = 5) was obtained by analyzing the sessile drop images using Drop Shape Analysis software affiliated with the goniometer. In order to measure roll-off angles, the stage tilt angle was gradually increased at a speed of 1 deg/sec from 0° until the point where the water droplet started to move along the stage.

For Scanning Electron Microscopy (SEM) analysis, specimens were mounted on aluminum stubs with carbon tabs prior to sputter-coating with 10 nm carbon and 3 nm platinum. SEM imaging was conducted at 10 kV accelerating voltage and a working distance of 10 mm using a field-emission SEM (Zeiss VP1555, Oberkochen, Germany). Other operating parameters were stipulated as follows: aperture size = 30.00 *μ*m, signal = SE2, gun vacuum = 1.30exp−10 Torr. Ten measurements of surface features were performed on each SEM image, from which the average and standard deviations values were derived.

## 3 Results and discussion

### 3.1 Leaf surface wettability

Special wetting features, namely high contact angles and strong water adhesion, were exhibited by the leaves of all three Australian native *Eucalyptus* species. This is similar to the findings on the petals of Rosa, *cv*. Bairage [11] and in sharp contrast with the wetting condition of the lotus leaf, *Nelumbo nucifera* characterized by high contact angles but negligible water retention.

**Fig. 1:**
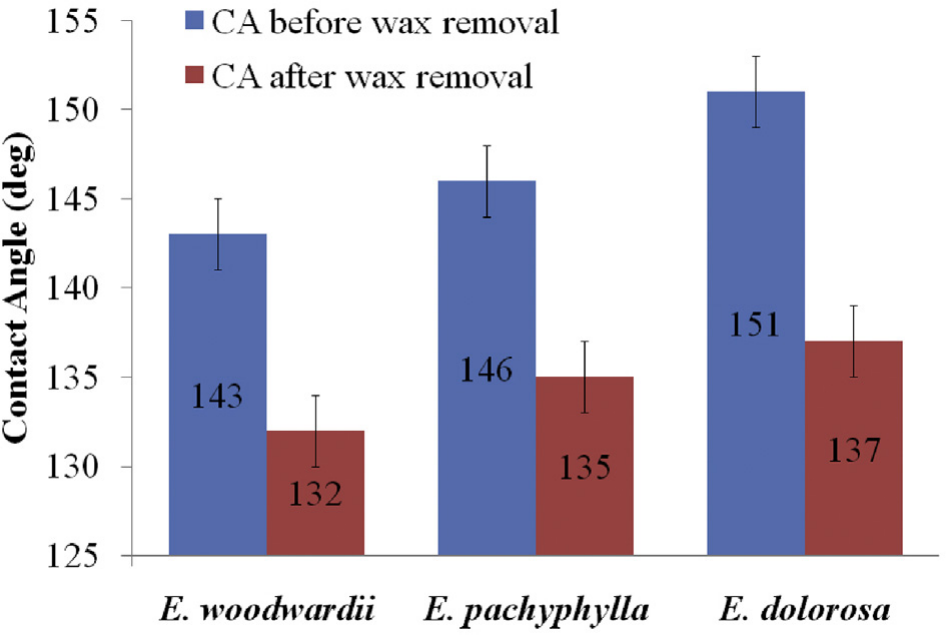
Experimental water contact angles of *Eucalyptus* leaves before and after wax removal.

The water contact angles of the study species (Fig. 1), when compared with that of lotus leaves (160.4 ± 0.7°) [21], can be considered as strongly hydrophobic. Given that the lotus is an emergent aquatic plant and the fact that the species of *Eucalyptus* thrive in drier environments, it is surprising that these *Eucalyptus* spp. exhibit such high levels of leaf hydrophobicity. Conversely, strong adhesion was observed on all three *Eucalyptus* species, before wax removal, between the water droplet and the leaf surface, with the droplet adhering firmly to the surface when it was gradually tilted from 0° to 180° (Figure not shown here). This adhesion may be due to a large contact area between the water and the leaf surface. Water retention on fresh leaves is believed to help maintain surface hydration following a rainfall or evening dew event by providing the leaf with an additional source of moisture, particularly since many *Eucalyptus* species are exposed to periods of low rainfall.

**Fig. 2:**
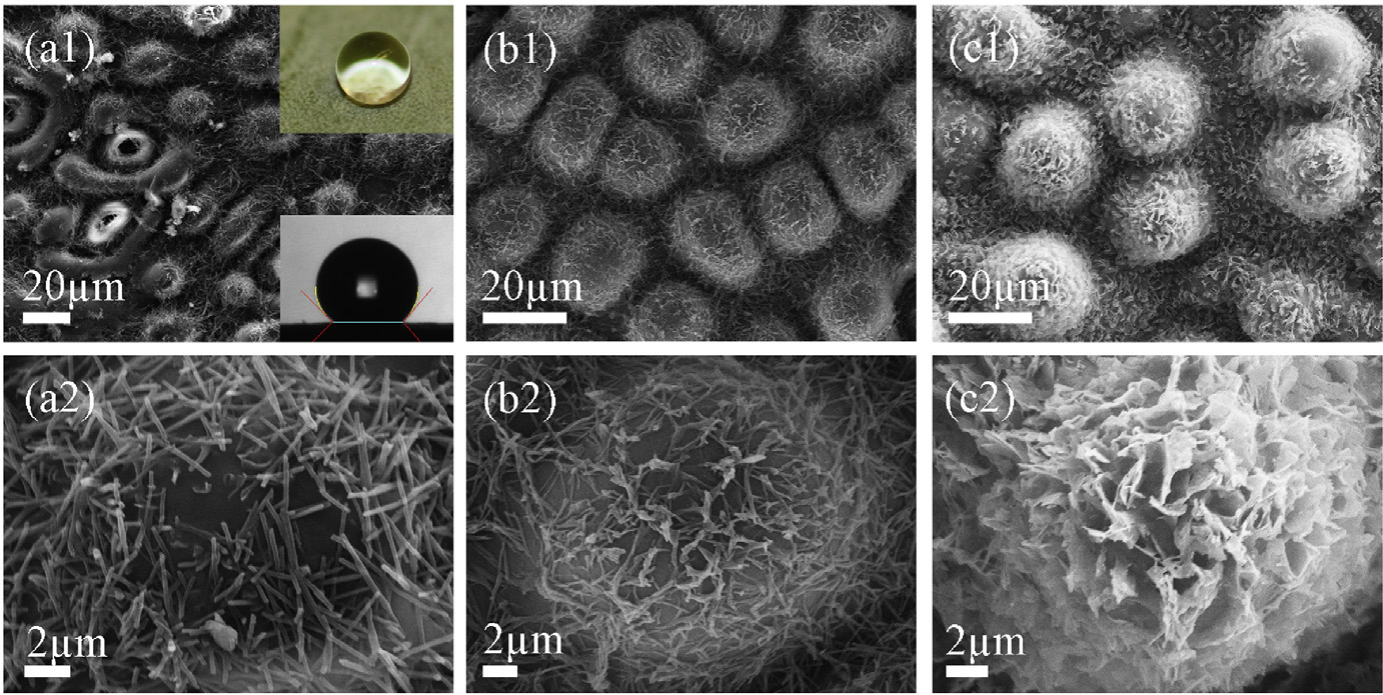
SEM images of three *Eucalyptus* leaves at two magnifications: (a) *Eucalyptus woodwardii* with optical images of water droplets shown in the inset, (b) *Eucalyptus pachyphylla*, and (c) *Eucalyptus dolorosa*. Scale bars: (1) 20 *μ*m and (2) 2 *μ*m.

The surface morphologies of three *Eucalyptus* leaves are shown in Fig. 2. Generally speaking, the entire surface of these leaves is covered by fine nanometer scale wax of different forms and microscopic papillae and stomata, forming at least two length scales of roughness. On the micrometer level, hemisphere-like papillae arranged in a quasi-hexagonal pattern appear on the leaf surfaces of all three *Eucalyptus* species. Different wax morphologies, however, can be identified at the nanometer level. Needle-like wax particles of approximately 5 *μ*m in length and 200 nm in diameter are aggregated on the leaf surface of *E. woodwardii* (Fig. 2a), while string-like wax covers the papillae of *E. pachyphylla* (Fig. 2b) and flake-like wax is found on *E. dolorosa* (Fig. 2c). These multi-scale structures have some morphological similarity to that of the lotus [21]. However, there is a major difference: for lotus leaves, the wax forms fine, nanometer-length short hairs positioned upright to the surface, while on these specific *Eucalyptus* leaves, wax features possess relatively large dimensions lying loosely over the surface.

In order to resolve the role that the wax structure plays in the observed wetting properties, water contact angles on these *Eucalyptus* leaves were measured and compared before and after wax removal (Fig. 1). For leaves free of wax, an average contact angle drop of 12 ± 1.7° was observed, which suggests a minor contribution to the contact angles coming from the wax and that the leaf micro-structures impart an inherent surface hydrophobicity.

**Fig. 3:**
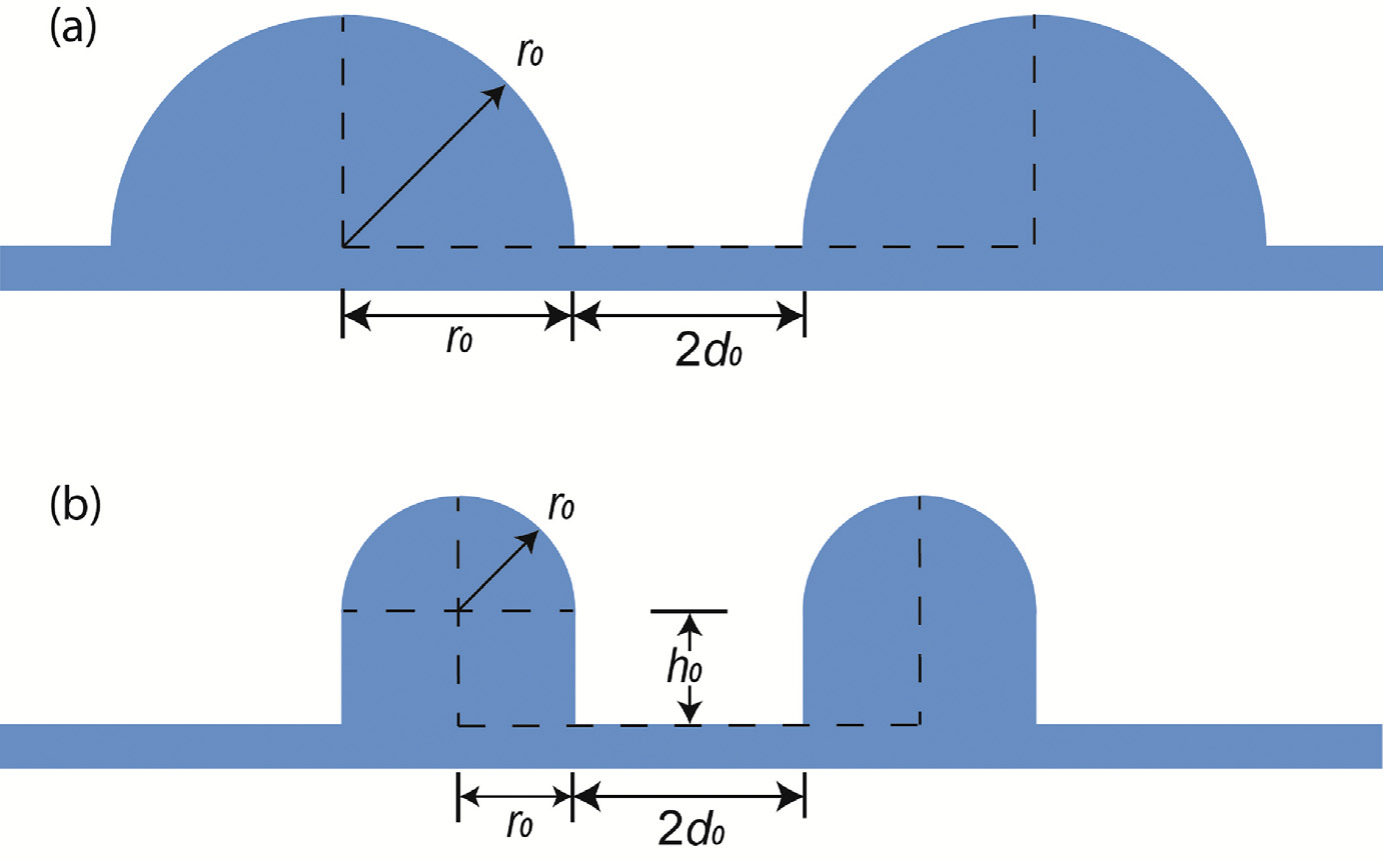
Generalized physical models of microscopic surface features for: (a) *Eucalyptus* leaf with parameters adopted from *Eucalyptus pachyphylla* and (b) lotus leaf *Nelumbo nucifera*.

The surface micro-structures of these *Eucalyptus* leaves appear to play a key role in determining water contact angles, as high contact angles are still maintained even after wax is removed. However, it should, be pointed out that the wax structures on the nanometer scale generally enhance the wetting robustness. For example, synthetic hierarchical surfaces with varying micro-structure pitch values and wax densities consisting of the nano-structures have been previously fabricated [11]. On these artificial surfaces water contact angles did not change substantially on 23 *μ*m pitch micro-structures across differing wax densities. However, there was a drop in the contact angle on a 105 *μ*m pitch micro-structure when less wax was deposited. Based on the above observation and analysis, wetting states of both the *Eucalyptus* and the lotus *Nelumbo nucifera* leaves may be modeled using their surface micro-structural parameters as input. The surface morphology of *Eucalyptus* leaves can be considered as multiple hemispheres sitting on a flat plane (Fig. 3a). Similarly, for the lotus [21], the leaf surface can be visualized as a plane containing many cylinders, each with a hemispherical cap (Fig. 3b). In both models, the radius of the hemisphere is denoted as *r*_0_, the height of the cylinder as *h*_0_, and the edge-to-edge distance between two neighboring micro-structures as *2d*_0_. When *h*_0_ equals zero, the two models are essentially identical.

The surface micro-structural parameters of both lotus and three *Eucalyptus* leaves, such as *r*_0_, *h*_0_ and *2d*_0_, were measured from SEM images and summarized in Table 1. It is understood that the arrangement of the micro-structured features on the leaf surface may affect the surface roughness, which in turn influences the contact angle. Two most common arrangements of the microscopic hemispheres, namely hexagonal and square patterns are considered here when calculating wetting properties. However, negligible difference (for example, <1% in contact angle) was observed from these two patterns in this study. Therefore, only data from the hexagonal pattern is reported here.

**Table 1:** Geometric parameters of micro-structures measured on the leaf surface of lotus *Nelumbo nucifera* and three *Eucalyptus* species.

Samples	Radius, *r_0_* (*μ*m)	Height, *h*_*0*_ (*μ*m)	Distance, *2d_0_* (*μ*m)
Lotus	5 ± 0.5	5 ± 0.5	10 ± 1.2
*E. woodwardii*	8 ± 0.8	0	14 ± 2.8
*E. pachyphylla*	9 ± 0.6	0	9 ± 2.8
*E. dolorosa*	10 ± 1.0	0	16 ± 5.4

### 3.2 Analysis of surface free energy

When a water droplet is gently deposited on a surface, based on the surface energy and roughness of the surface, one of the two classical wetting regimes can exist. In the homogeneous Wenzel wetting, water fills in the asperities of the micro-structure and maintains the maximum contact with the surface. As such, the contact angle is expressed as [12]

(1) $$\cos\theta_{W}=r\;\cos\theta_{Y}$$

where *θ*_W_ and *θ*_Y_ are the Wenzel and Young contact angles, respectively. The roughness factor, *r*, is defined as the ratio of the actual solid-liquid contact area to its projected area on the horizontal surface plane. Alternatively, the heterogeneous Cassie model, characterized by the presence of air pockets between water and the leaf surface [13], can be used to calculate the contact angles as

(2) $$\cos\theta_{C}=r_{f}f\;\cos\theta_{Y}+f-1$$

where *θ*_C_ and *θ*_Y_ are the Cassie and Young contact angles, respectively, *r*_*f*_ is the roughness ratio of the actual solid-liquid contact area and *f* is the fraction of the solid-liquid contact area projected on the surface plane over the total surface projection.

For this three-phase system, different surface energies exist for specific wetting scenarios. There are, therefore, energy barriers between various wetting states. A principle of energy minimization is generally applied to determine the final wetting state. That is, the liquid will wet the rough surface to the extent that the overall surface energy of the system is minimized. A prerequisite for taking the final state of minimum energy is that the system can overcome the energy barriers lying between different conditions in a certain environment, given that wetting is a continuous physical process. Thus it is possible for the system to reach equilibrium before the surface energy reaches minimum due to insurmountable energy barriers presented without any external disturbance. In order to analyze the possibility of transition between different wetting states, it is necessary to consider the corresponding Gibbs free energy analysis. The Gibbs free energy of a droplet on a surface can be described as [22, 23]

(3) $$G=\gamma_{LV}S_{LV}+\gamma_{SV}S_{SV}+\gamma_{LS}S_{LS}$$

where *γ*_*ij*_ is the surface energy between the interface *ij* (solid, liquid and vapor), and *S*_*ij*_ is the contact area of the interface *ij* (solid, liquid and vapor).

Before substituting with mathematic expressions for these interfacial areas, certain assumptions and reasonable approximations are required based on actuality: (a) the influence of gravity can be neglected, as the radius of a water droplet used here (5 *μ*L, ∼1.0 mm) is usually less than the capillary length (∼2.7 mm). Thus, the droplet can be deemed to be spherical; (b) the radius of a water droplet is significantly larger than the surface roughness, and the radius of the liquid-vapor interface meniscus, between surface reliefs, can be approximated to the radius of the water droplet. Consequently, the liquid volume of entering the asperities of the micro-structures can also be neglected, and the liquid-vapor interface meniscus can be taken as planar, parallel to the surface horizontal plane; (c) the base of the spherical droplet can be approximated as the projection of the liquid-solid contact on the surface plane; (d) the contact line tension and potential energy are negligible as their contributions to the total surface energy are insignificant. When relating to the droplet volume *V* and contact angle *θ*, the radius of the droplet *R* can be expressed as [22]

(4) $${R={\left(\frac{3V}{\pi }\right)}^{\frac{1}{3}}\left(2-3\;\cos\;\theta +\cos^{3}\;\theta \right)}^{-\frac{1}{3}}$$

In a composite wetting state, the interfacial areas are calculated using *r*_f_ and *f*, the parameters defining the Cassie relationship. *S_LV_* consists of two parts: the external spherical cap of the droplet and the liquid-vapor contact under the droplet.

(5) $$S_{LV}=2\pi R^{2}\left(1-\cos\;\theta \right)+\pi R^{2}\;\sin^{2}\;\theta \left(1-f\right)$$

*S*_*LS*_ only contains the liquid-solid contact area under the droplet.

(6) $$S_{LS}=\pi R^{2}\;\sin^{2}\;\theta \;r_{f}f$$

*S*_*SV*_ also consists of two parts: the solid-vapor contact under and outside the droplet, respectively. *S*_*SV-total*_ is introduced here as the total solid-vapor contact area before the droplet deposition. For a specific surface, *S*_*SV-total*_ is constant.

(7) $$S_{SV}=\left(S_{sv-total}-\pi R^{2}\;\sin^{2}\;\theta \;r\right)+\pi R^{2}\;\sin^{2}\;\theta \;\left(r-r_{f}f\right)$$

The Gibbs energy of a composite wetting system can be described by plugging Eqs. (4), (5), (6), and (7) into Eq. (3)

(8) $${G_{composite}=\gamma_{SV}S_{SV-total}+\gamma_{LV}\left(3V\right)}^{\frac{2}{3}}\pi^{\frac{1}{3}}{\left[F\left(\theta \right)\right]}^{-\frac{2}{3}}\left[2-2\;\cos\;\theta -\sin^{2}\;\theta \;F\left(f\right)\right]$$

with

(9) $$F\left(\theta \right)=2-3\;\cos\;\theta +\cos^{3}\;\theta$$

and

(10) $$F\left(f\right)=r_{f}f\;\cos\theta_{Y}+f-1$$

For a droplet with a volume *V* and a well-defined solid surface, the surface energy of the system changes with the contact angle *θ* and its specific wetting condition (in which these two parameters *r*_f_ and *f* can be determined). The surface roughness ratio, *r*, does not affect the value of *G*_composite_ due to its absence in Eq. (8). However, Eq. (8) is a universal equation that remains valid for both composite and wetted regimes. Similar to the conversion between Wenzel and Cassie relations, the function $F\left(f\right)=r_{f}f\;\cos\theta_{Y}+f-1$ for a composite regime can be simplified to $F\left(f\right)=r\;\cos\theta_{Y}$ for a wetted regime. Thus, the Gibbs energy for a wetted case (where there is no air trapped under the droplet and thus no liquid-vapor and solid-vapor contact areas), can also be obtained with the corresponding *F*(*f*) expression.

Supposedly the wetting transition from a composite case to a wetted case begins with gradual penetration of liquid into the asperities. Let *h* be the penetration depth, *r*_0_ be the micro-hemisphere base radius and *d*_0_ half of the edge-to-edge distance of the micro-hemispheres in a hexagonal arrangement. *h* can be reasonably approximated to *r*_0_ by $h=xr_{0}$, where *x* is in the range of (0, 1) for the model of the *Eucalyptus* spp., and *d*_0_ can be approximated to *r*_0_. The wetting parameters for a composite case can be expressed as

(11) $$r_{f}=\frac{2r_{0}}{2r_{0}-h}=\frac{2}{2-h}$$

and

(12) $$f=\frac{{\pi [{r_{0}}^{2}-(r_{0}-h)}^{2}]}{{\frac{\sqrt{3}}{2}(2r_{0}+2d_{0})}^{2}}=\frac{\pi (2x-x^{2})}{8\sqrt{3}}$$

while the wetted regime can be expressed as:

(13) $$r=1+\frac{\pi {r_{0}}^{2}}{{2\sqrt{3}\left(r_{0}+d_{0}\right)}^{2}}=1+\frac{\pi }{8\sqrt{3}}$$

The function *F*(*f*) can be rewritten when substituting Eqs. (11) and (12)

(14) $$F\left(f\right)=F\left(x\right)=\frac{4\pi x\;\cos\theta_{Y}-\left(2-x\right)\left(\pi +2\sqrt{3}\right)}{\left(2-x\right)\left(\pi +2\sqrt{3}+2\pi x\right)}$$

It is assumed here that the transition between a composite and wetted state involves gradual liquid penetration into the rough micro-structures. For each depth of penetration, there is a specific contact angle for the droplet in order to achieve the minimum surface energy. At the thermodynamic equilibrium, the contact angle *θ* equals the Cassie contact angle *θ*_*C*_ and Eq. (8) can be used to calculate the Gibbs surface energy

(15) $${G_{Cassie}=\gamma_{SV}S_{SV-total}+\gamma_{LV}\left(3V\right)}^{\frac{2}{3}}\pi^{\frac{1}{3}}{\left[F\left(\theta \right)\right]}^{\frac{1}{3}}$$

with

(16) $$F\left(\theta_{C}\right)=2-3\;\cos\;\theta +\cos^{3}\theta_{C}$$

In Eq. (15), both the first part on the right side, $\gamma_{SV}S_{SV-total}$ and ${\gamma_{LV}\left(3V\right)}^{\frac{2}{3}}\pi^{\frac{1}{3}}$ are positive and constant, the actual value of *G*_*Cassie*_ can be compared through *F*(*θ*_*C*_), given the exponent on *F*(*θ*_*C*_) is 1/3. In these calculations, 104^◦^ was adopted as the water contact angle on a flat surface covered by wax [22].

The value of *F*(*θ*_*C*_) decreases as the penetration depth *x* increases from nil to 0.75. At about 0.75, the minimum of *F*(*θ*_*C*_) is obtained (3.951), which indicates the minimum surface energy for this specific geometry and composite wetting. After 0.75, *F*(*θ*_*C*_) begins to increase slowly. This observation is similar to the case found on flat-top microscopic cylindrical pillars. When *x* reaches unity, liquid touches the bottom and the wetting scenario shifts from composite to wetted, for which the *F*(*θ*_*W*_) value can be calculated as 2.867 by employing Eqs. (13), (1), and (8). Subsequently, when a droplet is deposited gently on a surface characterized with microscopic hemispheres ($r_{0}=d_{0}$), liquid under the droplet simultaneously enters the asperities and reaches a thermodynamic equilibrium at *x* = 0.75 to achieve the minimum surface energy for the composite regime (with local pinning and external energy input neglected). Since the wetted case possesses lower surface energy than the composite case, a transition occurs when the vibration energy of the droplet or an external energy input can surpass the energy barrier, which can be calculated as the surface energy difference between the two states of x ≈ 0.75 and x ≈ 1. This method of energy barrier calculation agrees with a postulation that, on flat-top microscopic pillars, the energy barrier is the energy difference between the non-filling (Cassie) and complete-filling states [23].

**Fig. 4:**
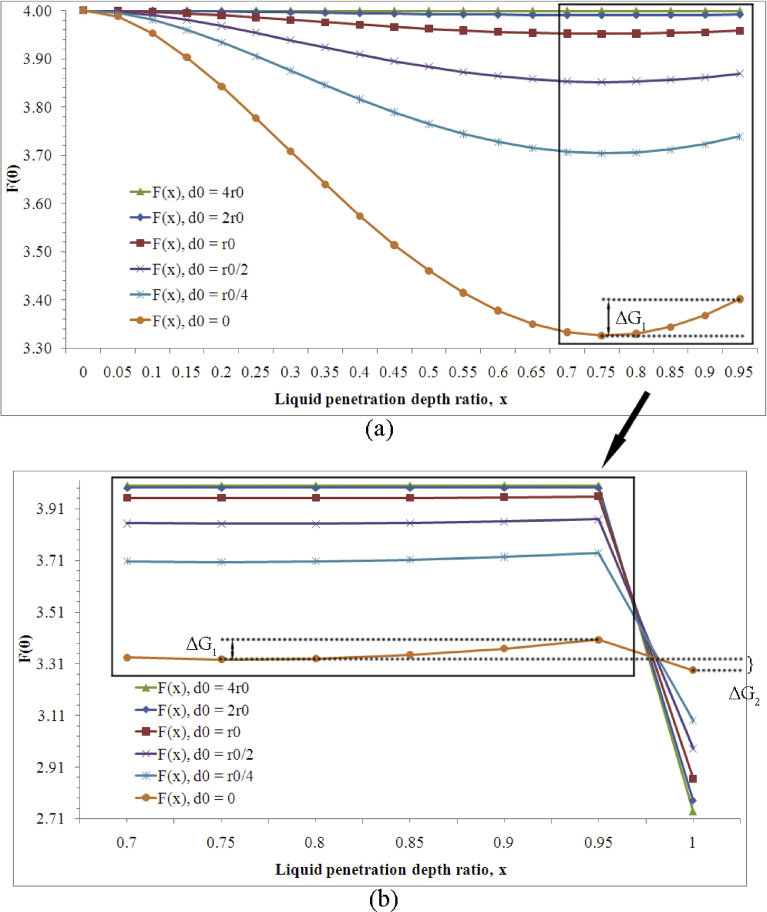
Influence of *d*_0_ on *F*(*θ*) values: (a) *F*(*θ*) at different penetration depths with different *d*_*0*_ values, (b) *F*(*θ*) at different penetration depths, 0.75 *≤ x* ≤ 1. The *F*(*θ*) values at the *x* range of (0.95, 1) are omitted. When *x* = 1, *F*(*θ*) = *F*(*θ*_*W*_).

The effect of edge-to-edge distance on the surface energy is studied by changing the value of *d*_0_ based on a constant *r*_0_, and the calculated *F*(*θ*_*C*_) values are shown in Fig. 4. A lower minimum surface energy is obtained at a smaller depth for a smaller *d*_0_. In addition, a larger energy barrier (marked as Δ*G*_1_ in Fig. 4a), which acts as an indicator of hydrophobic stability, exists for the wetting transition for a smaller *d*_0_. Therefore, a smaller *d*_0_, such as *d*_0_ = 0, is favored to maintain a composite wetting state.

A portion of the *F*(*θ*_*C*_) curve in Fig. 4a is expanded with an extension of the *F*(*θ*) values at wetted regimes. After a minimum surface energy is reached, *F*(*θ*) value (or accordingly the surface energy) increases gradually with further liquid penetration before complete wetting of the asperities. Although not shown in Fig. 4b, the *F*(*θ*) values at the *x* range of (0.95, 1) can be reasonably postulated as being slightly higher than the values acquired at *x* = 0.95. The surface energy difference between the composite state (with a minimum surface energy) and the wetted state, marked as Δ*G*_*2*_ in Fig. 4b, can be considered as a transition potential or tendency. A larger energy difference, accompanied with a smaller barrier, reflects an easier composite-wetted transition for a larger *d*_0_.

The energy barrier Δ*G*_*1*_ and energy potential Δ*G*_*2*_ for the wetting transition for different *d*_0_ values are calculated using Eq. (15). The volume of the water droplet is taken as 5 *μ*L. The energy barrier is calculated as the difference between the lowest energy and the energy at *x* = 0.99, just before the wetting transition. The energy potential is calculated as the difference between the lowest energy at a composite state and the lowest energy at a wetted state. The results are shown in Table 2.

**Table 2:** Calculated energy barriers and energy potentials for different *d*_*0*_.

*d*_0_	ΔG_1_ Energy barrier, nJ	ΔG_2_ Energy potential, nJ
0	7.33	4.05
*r*_0_/4	3.11	58.84
*r*_0_/2	1.56	83.24
*r*_0_	0.52	104.19
2*r*_0_	0.065	116.83
4*r*_0_	0.013	122.19

When *d*_0_ decreases from *r*_0_ to *r*_0_/4, the energy barrier increases from 0.52 nJ to 3.11 nJ, indicating enhanced hydrophobic stability or robustness. When *d*_0_ = 2*r*_0_, the energy barrier is close to nil and a droplet deposited gently on such a surface will spontaneously enter the roughness to the thermodynamically-favored wetted state.

In the model for the lotus, the existence of *h*_0_ leads to further extension of the penetration depth *x* at the range of [1, 1 + *h*_0_/*r*_0_]. With *d*_0_ being approximated to *r*_0_, the wetting parameters for a composite case, in which the liquid level in the asperities is below the microscopic hemisphere base, can be expressed as

(17) $$r_{f}=\frac{2\pi {r_{0}}^{2}+2\pi {r_{0}}^{2}\left(x-1\right)}{\pi {r_{0}}^{2}}=2x$$

and

(18) $$f=\frac{\pi {r_{0}}^{2}}{{\frac{\sqrt{3}}{2}\left(2r_{0}+2d_{0}\right)}^{2}}$$

while the wetted regime be expressed as:

(19) $$r=1+\frac{\pi {r_{0}}^{2}+2\pi r_{0}h_{0}}{{\frac{\sqrt{3}}{2}\left(2r_{0}+2d_{0}\right)}^{2}}$$

**Fig. 5:**
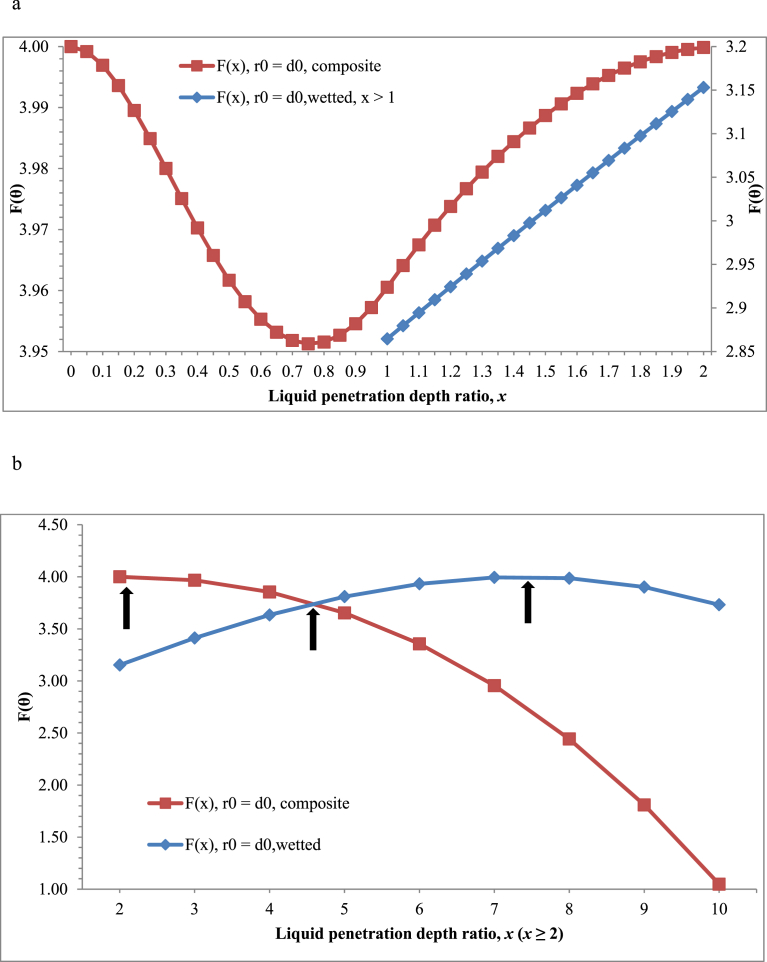
*F*(*θ*_*C*_) values for the lotus model at different penetration depths of *x*: (a) 0 *≤ x* ≤ 2, the composite state is marked by red (left vertical axis) and the wetted state is marked by blue (right vertical axis); (b) 2 *≤ x* ≤ 10, the composite state is marked by red and the wetted state is marked by blue. Three black arrows denote turning points on surface energy at *x* = 2.05, 4.6 and 7.4, respectively.

The *F*(*θ*_*C*_) values with *x* at the range of [1, *h*_0_) can be derived by combining Eqs. (9) and (10) for the free surface energy expressions, and Eqs. (17), (18), and (19) for the roughness factors. In Fig. 5a, the curve is the same as the *Eucalyptus* model in the range of [0, 1). After further liquid penetration (x ≥ 1), *F*(*θ*_*C*_) maintains increase with a gradually decreasing slope. At the same range, the *F*(*θ*_*W*_) value (corresponding to the wetted surface energy and marked in blue), increases with *x* at a constant slope of $\pi /\left(4\sqrt{3}\right)$. At large depths of *x* (continued in Fig. 5b), the *F*(*θ*_*C*_) value reaches a maximum and then declines gradually even when *x* = 10. The *F*(*θ*_*W*_) value increases with *x* and reaches a maximum at a much larger depth and then declines slowly. An intersection is found for these two cures at *x* = 4.6. After this point, the composite regime is more thermodynamically-favored than the wetted regime.

It should be noted that Eq. (15) can only be used when the thermodynamic equilibrium is reached and the Cassie relationship is applicable. After *x* = 2.05, the *F*(*f*) value in Eq. (10) exceeds unity and no solutions for the contact angle will be found to fulfill the equation of *F*(*f*) = cos*θ*_C_. The same issue occurs for calculating the minimum surface energy for a wetted scenario using the Wenzel relation when *x* > 7.4. After these two specific points, only the original Eq. (8) can be adopted for the overall surface energy calculation and the results stand for situations when a thermodynamic equilibrium cannot be reached.

Table 3 reveals that greater energy barriers and lower energy potentials are obtained for greater *h*_0_ values. The change of *h*_0_ from 0 to *r*_0_ significantly elevates the energy barrier from 0.86 to 4.22 nJ and decreases the energy potential from 104.16 to 74.28 nJ. When *h*_0_ > 1, all wetting conditions with the penetration depth of *x* > 2*r*_0_ face the same maximum energy barrier (4.22 nJ) arising from this specific geometry. Before *x* reaches 7.4*r*_0_ and after the energy barrier is overcome, a wetted regime with lower surface energy will be adopted by the system. However, neither a composite regime nor a wetted regime will be thermodynamically-favored with a liquid penetration deeper than 7.4*r*_0_. In this case, the droplet will adopt the composite state with a much smaller *x* at a thermodynamic equilibrium.

**Table 3:** Calculated energy barriers and energy potentials for different *h*_0_.

*h*_0_	Δ*G*_*1*_ Energy barrier, nJ	Δ*G*_*2*_ Energy potential, nJ
0	0.86	104.16
*r*_*0*_/4	2.24	96.30
*r*_*0*_/2	3.28	88.65
3*r*_*0*_/4	3.88	81.37
*r*_*0*_	4.22	74.28

The geometrical parameters presented in Fig. 3 can be adopted to compare the energy barriers and energy potentials of wetting on *Eucalyptus* and lotus leaves. For *Eucalyptus pachyphylla*, *r*_0_ = 2*d*_0_ = 9 *μ*m and *h*_0_ = 0, the energy barrier and energy potential are 1.56 nJ and 83.24 nJ, respectively. For lotus, 2*r*_0_ = 2*d*_0_ = 2*h*_0_ = 10 *μ*m and these two values are 4.22 nJ and 74.28 nJ, respectively. The energy barrier for a wetting transition on a lotus leaf is nearly three times that of a *Eucalyptus pachyphylla* leaf. The liquid-solid contact area, *S*_*LS*_, is also compared between these two models, as the contact area is expected to be strongly associated with the water-leaf adhesion. Given that the minimum surface energies at a composite state for the two models are obtained at a similar liquid penetration depth ratio (*x* ≈ 0.75, Figs. 4 and 5a), the radii of a 5 *μ*L water droplet on these two models are calculated using Eqs. (4), (11), and (12). For *Eucalyptus* and lotus models, the radii are found to be 1.074 mm and 1.065 mm, respectively. After substituting Eq. (6) with the radii, the liquid-solid contact areas were calculated to be 0.908 mm^2^ and 0.298 mm^2^, respectively. Therefore, for a water droplet of 5 *μ*L, the liquid-solid contact area on the *Eucalyptus* spp. is three times that on the lotus leaf. Even though several assumptions and approximations have been made on the energy analysis and calculations, the results can be used to describe qualitatively the stability of hydrophobicity and the surface adhesion for two different kinds of hydrophobic leaves in nature. Further work will be focused on verification of these results by experimental data on micro-fabricated surfaces and the wetting prediction of multiple roughness on these models.

It was the first time that geometrical models were proposed based on the surface morphologies of *Eucalyptus* leaves and lotus leaves to analyze quantitively two different wetting phenomena observed in nature. Energy potential for wetting transition was also identified at the first place in the surface free energy curve. This methodology of free energy analysis using geometrical models could be useful for wettability calculations and evaluation of surfaces on the micro-meter level roughness. Further work will be focused on verification of these results by experimental data on micro-fabricated surfaces and the wetting prediction of multiple roughness levels on these models.

## 4 Conclusions

Considerable hydrophobicity and strong adhesion were found on the leaves of three Australian native *Eucalyptus* species. Multi-scale roughness, namely, micro-structural bumps and nano-structural wax, was revealed by SEM on these leaf surfaces. Physical models were proposed based on the surface morphologies to gain a deeper understanding of the wetting mechanisms of *Eucalyptus* leaves in comparison to the lotus leaf, *Nelumbo nucifera*. Surface energy analysis on these two models shows the changes of minimum surface energy with stepwise liquid penetration into the asperities of the microscopic roughness. Lower minimum surface energy was found on a smaller edge-to-edge distance of the micro-structures in both models. The wetting transition from a composite state to a wetted state was quantitatively identified using energy barrier and energy potential as criteria. Greater cylindrical length below the microscopic hemisphere in the lotus model increases the energy barrier for the wetting transition, which is beneficial for hydrophobic stability. The strong water adhesion on *Eucalyptus* leaves was also explained by a relatively larger liquid-solid contact area than for lotus leaves.

This study represents a novel attempt to understand the unique wetting behavior of *Eucalyptus* leaves using a hemisphere-top model, given the facts that these *Eucalyptus* species present a different wetting scenario from the famous “lotus effect”. These findings could be potentially useful for guiding the design of man-made surfaces with tailored wetting properties. The potential applications behind the *Eucalyptus* model will be but not limited to micro-liter mass transport or microfluidic devices instead of superhydrophobicity and self-cleaning.

## Declarations

### Author contribution statement

Hua Guo: Conceived and designed the experiments; Performed the experiments; Analyzed and interpreted the data; Contributed reagents, materials, analysis tools or data; Wrote the paper.

Zonghan Xie: Analyzed and interpreted the data; Contributed reagents, materials, analysis tools or data; Wrote the paper.

Zhong-Tao Jiang: Conceived and designed the experiments; Contributed reagents, materials, analysis tools or data.

Jeremy Shaw: Performed the experiments; Contributed reagents, materials, analysis tools or data.

Kingsley Dixon: Contributed reagents, materials, analysis tools or data.

Chun-Yang Yin: Performed the experiments.

Xuemei Liu: Analyzed and interpreted the data; Wrote the paper.

### Funding statement

This work was supported by the Edith Cowan University Postgraduate Research Scholarship.

### Competing interest statement

The authors declare no conflict of interest.

### Additional information

No additional information is available for this paper.
